# Plasticity in PYD assembly revealed by cryo-EM structure of the PYD filament of AIM2

**DOI:** 10.1038/celldisc.2015.13

**Published:** 2015-06-23

**Authors:** Alvin Lu, Yang Li, Qian Yin, Jianbin Ruan, Xiong Yu, Edward Egelman, Hao Wu

**Affiliations:** 1 Department of Biological Chemistry and Molecular Pharmacology, Harvard Medical School, Boston, MA, USA; 2 Program in Cellular and Molecular Medicine, Boston Children's Hospital, Boston, MA, USA; 3 Department of Biochemistry and Molecular Genetics, University of Virginia, Charlottesville, VA, USA

**Keywords:** AIM2 PYD filament, cryo-EM, helical reconstruction, plasticity, inflammasome, nucleated polymerization, ASC-dependent inflammasome, Death Domain interaction

## Abstract

Absent in melanoma 2 (AIM2) is an essential cytosolic double-stranded DNA receptor that assembles with the adaptor, apoptosis-associated speck-like protein containing a caspase recruitment domain (ASC), and caspase-1 to form the AIM2 inflammasome, which leads to proteolytic maturation of cytokines and pyroptotic cell death. AIM2 contains an N-terminal Pyrin domain (PYD) that interacts with ASC through PYD/PYD interactions and nucleates ASC^PYD^ filament formation. To elucidate the molecular basis of AIM2-induced ASC^PYD^ polymerization, we generated AIM2^PYD^ filaments fused to green fluorescent protein (GFP) and determined its cryo-electron microscopic (cryo-EM) structure. The map showed distinct definition of helices, allowing fitting of the crystal structure. Surprisingly, the GFP-AIM2^PYD^ filament is a 1-start helix with helical parameters distinct from those of the 3-start ASC^PYD^ filament. However, despite the apparent symmetry difference, helical net and detailed interface analyses reveal minimal changes in subunit packing. GFP-AIM2^PYD^ nucleated ASC^PYD^ filament formation in comparable efficiency as untagged AIM2^PYD^, suggesting assembly plasticity in both AIM2^PYD^ and ASC^PYD^. The DNA-binding domain of AIM2 is able to form AIM2/DNA filaments, within which the AIM2^PYD^ is brought into proximity to template ASC^PYD^ filament assembly. Because ASC is able to interact with many PYD-containing receptors for the formation of inflammasomes, the observed structural plasticity may be critically important for this versatility in the PYD/PYD interactions.

## Introduction

Inflammasomes are cytosolic supramolecular complexes assembled in response to pathogen- and other damage-associated stimuli to activate pro-inflammatory responses through the maturation of interleukin 1β (IL-1β) and interleukin 18 (IL-18) [1]. They typically consist of a sensor component, after which the names of the inflammasomes are given, an adaptor component and an effector component such as caspase-1 and possibly caspase-11 (mouse), caspase-4 (human) and caspase-5 (human) [2]. Upon stimulation, the sensor components may convert from an auto-inhibited state and oligomerize to recruit an adaptor protein, which in turn recruit and activate the caspase zymogens through auto-proteolysis. Activated caspases then target the pro-forms of IL-1β and IL-18 for proteolytic maturation. These pro-inflammatory cytokines are released outside of the cell to trigger the downstream interleukin receptor pathway and induce the transcription of pro-inflammatory genes. Another possible outcome of inflammasome activation is pyroptotic cell death, which strengthens the inflammatory response.

Canonical inflammasome sensors can be classified into two classes, nucleotide-binding and oligomerization domain (NOD)-like receptors (NLRs) and absent in melanoma 2 (AIM2)-like receptors (ALRs). NLRs contain an N-terminal domain such as caspase recruitment domain (CARD), Pyrin domain (PYD) or baculoviral IAP repeat (BIR) domain for the recruitment of other inflammasome components. Taking the initials from these recruitment domains, NLRs are named as NLRP, NLRC and NLRB (also known as NAIP), respectively. NLRs also consist of a central NOD and a C-terminal leucine-rich repeat (LRR) domain [3]. NLR activation is thought to be the result of binding of the LRR domain to stimuli or partner molecules leading to a conformational change in the NOD-LRR region, which releases the auto-inhibition of the NOD domain. In the human genome, there are 22 putative NLRs [4].

ALRs comprise an N-terminal PYD and C-terminal HIN domains for double-stranded DNA (dsDNA) recognition. The two known ALRs, AIM2 and IFI16, are able to assemble functional inflammasomes [5, 6]. AIM2 was first identified in a proteomic-genomic screen that linked its role to IL-1β maturation [7–10]. The orthopoxvirus vaccinia virus and the beta-herpesvirus murine cytomegalovirus have been shown to directly trigger AIM2 activation in murine macrophages [6, 7]. While AIM2 assemble inflammasomes in the cytosol, IFI16 has been shown to shuttle between the cytosol and the nucleus, and functions as a nuclear pathogen sensor in Kaposi’s sarcoma-associated herpesvirus -infected endothelial cells [5].

The HIN domain is composed of tandem oligonucleotide/oligosaccharide binding (OB) folds, as first revealed from the crystal structures of IFI16 HIN-A and HIN-B [11]. Each OB fold conforms to the canonical OB domain architecture, which is a closed β-barrel with five twisted β-strands and an α-helix connecting the β3 and β4 strands [12]. Later, the structures for human AIM2 HIN and IFI16 HIN-B in complex with dsDNA were determined to show that the OB lobes and the in-between linker directly bind the dsDNA phosphate backbone [13]. Crystal structure of the mouse AIM2 in complex with dsDNA and related mutagenesis experiments showed the similar binding mode [14, 15]. The HIN domain of AIM2 was also shown to interact directly with the PYD of AIM2 to exert an auto-inhibition effect, and binding to dsDNA liberates PYD for inflammasome assembly [13, 16, 17]. The mouse ALR p202 without the N-terminal PYD is a specific inhibitor of AIM2 inflammasome activation [18].

ALRs and PYD-containing NLRPs recruit the bipartite adaptor ASC that contains both an N-terminal PYD and a C-terminal CARD. While the PYD of ASC (ASC^PYD^) interacts with PYD in ALR and NLR sensors, the CARD of ASC (ASC^CARD^) recruits CARD-containing effectors such as caspase-1. We and others have shown that ASC^PYD^ forms filamentous structures [17, 19, 20] with helical symmetry, which has emerged to be a conserved assembly mechanism for proteins in the death domain (DD) superfamily that includes both PYD and CARD [21]. The helical assembly involves three conserved types of asymmetric interactions. We found that ASC-dependent inflammasomes are formed through two-step nucleated polymerization [17]. For the AIM2 inflammasome, upon binding of the HIN domain to dsDNA, the AIM2^PYD^ domain clusters to nucleate the formation of ASC^PYD^ filaments. Analogously, activated NLRPs contain clustered PYD that templates ASC^PYD^ filament polymerization. We determined the ASC^PYD^ filament structure through cryo-electron microscopy (cryo-EM) at a resolution of ~3.8 Å, which revealed the detailed molecular interactions between the ASC^PYD^ subunits [17]. Subsequently, the resulting clustered ASC^CARD^ domain outside of the central ASC^PYD^ filament acts to nucleate caspase-1 filaments through CARD/CARD interactions, leading to signal amplification from the AIM2 sensor, to the ASC adaptor and then to caspase-1 [17]. The formation of caspase-1 filaments initiates proximity-driven dimerization and auto-proteolysis, and hence caspase activation.

How AIM2^PYD^ nucleates ASC^PYD^ filament formation is currently unclear. To resolve this question, we used cryo-EM to determine the filament structure of a GFP-fused AIM2^PYD^. The structure surprisingly revealed that the GFP-AIM2^PYD^ filament is a 1-start helix with helical parameters distinct from those of the 3-start ASC^PYD^ filament. However, despite the apparent symmetry difference, helical net and detailed interface analyses both showed minimal changes in subunit packing. The apparent ability of PYDs to adopt different helical symmetries indicates plasticity in their assembly. All ALRs and most members of the large NLR family have a PYD, which use the single adaptor ASC for inflammasome formation. As the ALR and NLR PYDs do not share high levels of sequence identity, there might be slight differences in their intrinsic filament forming symmetry, and yet all these PYDs have to interact with the same ASC^PYD^. In this context, the observed plasticity must be important for this versatility in the PYD/PYD interactions. It is remarkable that a significant difference in helical symmetry only results in small local changes in subunit packing in these PYD filaments. Given the similar mode of interactions utilized for the entire DD superfamily, the plasticity in symmetry and interaction may be general characteristics of the superfamily and likely underlie the wide evolutionary success of these domains in innate immunity.

## Results

### Structure determination of the GFP-AIM2^PYD^ filament by cryo-EM

**Figure 1:**
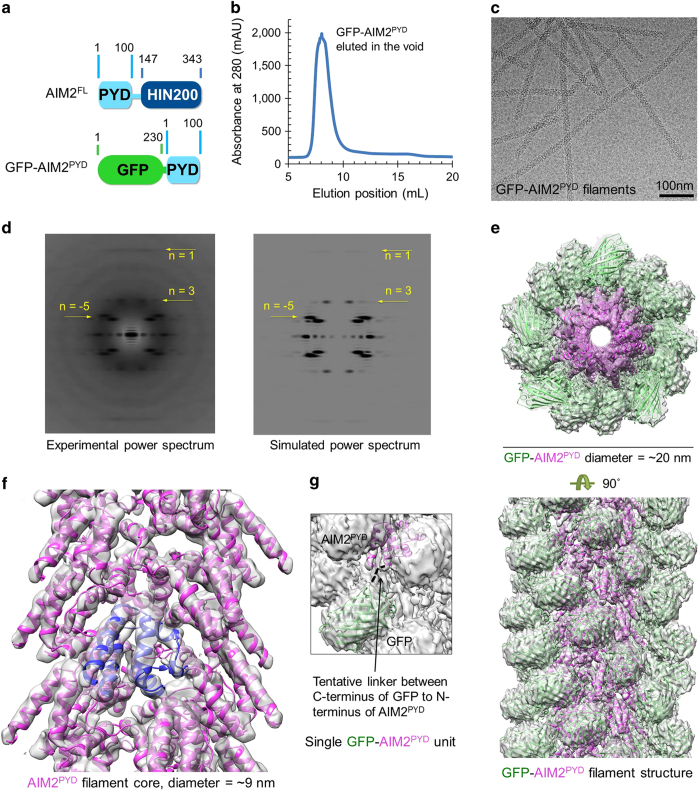
Cryo-EM structure determination of the GFP-AIM2^PYD^ filament. (**a**) Domain organizations of AIM2 and its PYD construct used for cryo-EM. (**b**) Gel filtration profile of the GFP-AIM2^PYD^ protein on a Superdex 200 10/300 size-exclusion column, showing elution at the void position. (**c**) A representative cryo-EM image. (**d**) Comparison of experimental and simulated power spectra. A few indexed layer lines are labeled. (**e**) Helical reconstructed cryo-EM map in two orientations of the GFP-AIM2^PYD^ filament fitted with atomic models of GFP (green, PDB ID: 1EMA) and partially refined AIM2^PYD^ (magenta, starting PDB ID: 3VD8). (**f**) A side view of the cryo-EM map of the AIM2^PYD^ filament core with the GFP region removed, fitted with the partially refined AIM2^PYD^ model. A single subunit is shown in blue. (**g**) A close-up view of one GFP-AIM2^PYD^ subunit, showing that the C-terminus of GFP is close to the N-terminus of AIM2^PYD^.

AIM2 contains an N-terminal PYD and a C-terminal dsDNA-binding HIN domain (Figure 1a). The AIM2^PYD^ could be over-expressed in *E. coli* but was hardly soluble, likely due to formation of extensive filamentous aggregates. To overcome this problem, we first tried to express AIM2^PYD^ as a His-MBP fusion in a monomeric form, followed by removal of the His-MBP tag to allow AIM2^PYD^ polymerization. However, cleaved, untagged AIM2^PYD^ immediately bundled (Supplementary Figure S1A) and quickly precipitated. We therefore reasoned that a non-cleavable tag which still allows filament formation is required for obtaining soluble filaments for cryo-EM studies. We fused AIM2^PYD^ to an N-terminal non-cleavable His-GFP tag with only a two-residue linker (Figure 1a). GFP-AIM2^PYD^ was purified by Ni-NTA affinity chromatography (Supplementary Figure S1B) followed by size-exclusion chromatography (SEC). It formed soluble aggregates as shown by elution in the void fractions of SEC (Figure 1b). Cryo-EM images showed that GFP-AIM2^PYD^ mostly formed filaments of limited lengths, from ~200 nm to ~1 μm (Figure 1c), which likely contributed to their solubility. The diameter of the filaments is ~20 nm.

Starting with an averaged power spectrum (Figure 1d), it was evident that many different helical symmetries were possible [22]. The correct helical symmetry was determined by trial-and-error, searching for a solution using the iterative helical real space reconstruction method [23] that yielded recognizable secondary structures [24]. The symmetry that was found, a rise of 6.0 Å and a right-handed rotation of 138.9° per subunit, generated a reconstruction where the PYD was seen to be largely α-helical. The AIM2^PYD^ subunit in the filament shows a six-helix bundle structure that is highly similar to the AIM2^PYD^ crystal structures in isolation [16, 19]. Thus, the reconstruction was taken to be correct. The calculated power spectrum from the reconstructed volume is consistent with the observed averaged power spectrum (Figure 1d).

In the filament, AIM2^PYD^ forms the core while the GFP tag packs tightly outside (Figure 1e; Supplementary Video 1). The AIM2^PYD^ crystal structure (PDB ID: 3VD8) can be readily fit into the central density (Figure 1f). Only minor adjustments of the individual helices were necessary, which were performed using the real space fitting tool in Coot [25]. In contrast to the clearly visible secondary structures in the AIM2^PYD^ region, the individual GFP density is essentially an ellipsoid that matches the size of a GFP molecule (PDB ID: 1EMA). We rotated the GFP molecule along its longer axis to place its C-terminus close to the N-terminus of AIM2^PYD^ to which the GFP is fused (Figure 1g). There is no clear density from the C-terminus of GFP to the fused N-terminus of AIM2^PYD^, suggesting flexibility between these two entities and explaining the much poorer resolution for the GFP tag. Estimation of resolutions by Fourier shell correlation between the densities and the fitted molecules gave ~5.0 Å for the AIM2^PYD^ region (Supplementary Figure S1C) and ~11 Å if GFP is included (Supplementary Figure S1D). The GFP density alone is probably resolved to only ~20 Å. The partially ordered GFP contributes to the doubling of the diameter of these filaments compared with the AIM2^PYD^ core, which is ~9 nm. Consistent with the estimation by Fourier shell correlation, the calculated model volumes filtered to 5 or 6 Å resolution showed features comparable to the experimental volume (Supplementary Figure S1E).

### The GFP-AIM2^PYD^ filament has a helical symmetry different from the ASC^PYD^ filament but retains the ability to nucleate ASC^PYD^ filament formation

**Figure 2:**
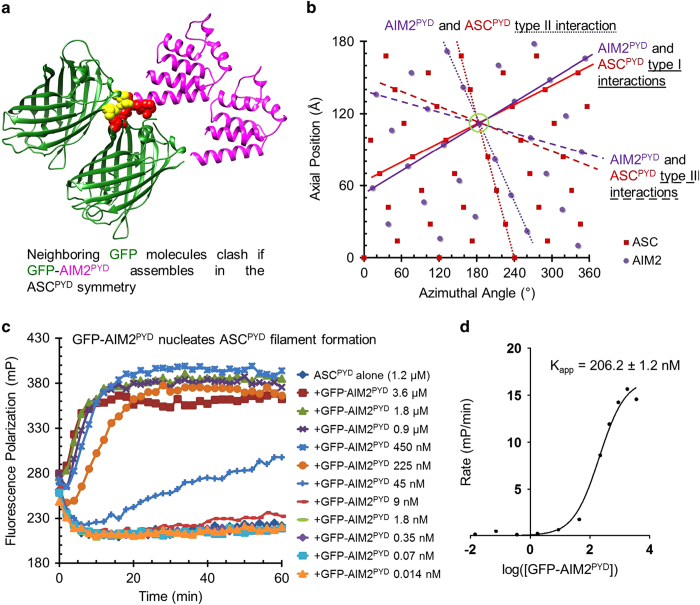
The GFP-AIM2^PYD^ filament has a different helical symmetry from ASC^PYD^ but retains the ability to nucleate ASC^PYD^ filaments. (**a**) GFP-AIM2^PYD^ subunits in the ASC^PYD^ filament symmetry showing steric clash in the GFP region (red and yellow). (**b**) Superimposed helical net plots of GFP-AIM2^PYD^ and ASC^PYD^ filaments showing similar orientations of the interactions despite the different symmetries. (**c**) Potentiation of ASC^PYD^ filament formation by GFP-AIM2^PYD^. (**d**) Initial slopes (mP/min) of (**c**) were plotted against the log of the concentrations (nM) of the added GFP-AIM2^PYD^ to extrapolate an apparent dissociation constant (*K*_app_) of the GFP-AIM2^PYD^/ASC^PYD^ interaction.

Given that AIM2^PYD^ and the full-length AIM2 in complex with dsDNA nucleate ASC^PYD^ filament formation [17], it was surprising that the AIM2^PYD^ filament has an apparent, almost completely different symmetry from our previously determined ASC^PYD^ filament structure. The latter is a 3-start helical assembly with C3 point group symmetry, having a right-handed rotation of ~52.9° and an axial rise 13.9 Å per subunit along each of the 3-start strands [17]. As the total axial rise per subunit of the ASC^PYD^ filament would be 13.9/3=4.6 Å, in comparison with 6.0 Å for the GFP-AIM2^PYD^ filament, within the same filament length, there would be (6.0−4.6)/6.0=23% fewer subunits in the GFP-AIM2^PYD^ filament. As the diameters of the ASC^PYD^ filament and the GFP-AIM2^PYD^ core are almost identical, at~9 nm, it dictates that the latter is packed significantly less densely, likely due to the fused GFP. If the modeled GFP-AIM2^PYD^ subunits are placed into a filament based on the ASC^PYD^ filament symmetry, one GFP molecule would be in a clash with an adjacent GFP molecule (Figure 2a). This exercise suggests that to accommodate the fairly large GFP tag (MW=28.1 kDa), the intrinsic AIM2^PYD^ helical filament may have been altered, resulting in less dense packing. The altered packing may have also destabilized the AIM2^PYD^ filament so that the GFP-AIM2^PYD^ filaments are shorter and more soluble than the AIM2^PYD^ filament without a large fusion tag, allowing *in vitro* reconstitution and cryo-EM structure determination.

We asked whether the difference in symmetry led to completely different packing in the AIM2^PYD^ and the ASC^PYD^ filaments. To compare the packing arrangements, we generated helical net plots for both filaments, in which each dot represents the location of a subunit, and aligned them using a subunit near the center as a reference (Figure 2b, and Supplementary Figures S2A and B). The aligned helical net plots showed that, despite the completely different symmetry, the locations of subunits in AIM2 and ASC filaments are quite similar, suggesting comparable spatial packing arrangements. In the ASC^PYD^ filament structure, three types of interactions mediate the assembly, with type I interactions for intrastrand contacts, and type II and III interactions for interstrand contacts. In the AIM2^PYD^ filament structure, the lines connecting these interactions differ minimally from those in the ASC^PYD^ filament, further corroborating the similar subunit packing contacts in both filaments.

The altered symmetry and tweaked packing in the GFP-AIM2^PYD^ filament suggest plasticity in the assembly and tolerance to variability. Our previous cryo-EM reconstruction of the ASC^PYD^ filament has already indicated intrinsic variability, as shown by an approximate Gaussian distribution of rotation angles per subunit from 51.5 to 54.5° [17]. However, the magnitude of change observed here between the GFP-AIM2^PYD^ filament structure and the ASC^PYD^ filament structure is much larger. To determine whether GFP-AIM2^PYD^ filaments with altered symmetry could still nucleate ASC^PYD^ filaments, we utilized a fluorescence polarization assay to monitor enhanced polarization due to filament formation and reduced motion. ASC^PYD^ was expressed as a fusion to the large solubility tag MBP (MBP-ASC^PYD^), which allowed purification of monomeric MBP-ASC^PYD^ on a size-exclusion column. The protein was then labeled by the Alexa568 fluorophore via an engineered C-terminal Cys residue using maleimide chemistry. The polymerization reaction was initiated by addition of the TEV protease to remove the MBP tag, upon which ASC^PYD^ spontaneously formed filaments at a very slow rate. Inclusion of sub-stoichiometric amounts of GFP-AIM2^PYD^ greatly enhanced the rate of ASC^PYD^ polymerization (Figure 2c). The apparent affinity for GFP-AIM2^PYD^ to promote ASC^PYD^ filament formation was determined to be ~0.21 μM by plotting the initial rates of ASC^PYD^ filament formation against the concentrations of the GFP-AIM2^PYD^ seeds (Figure 2d). We also repeated a polymerization experiment published in our previous study [17] in which MBP-AIM2^PYD^ and labeled MBP-ASC^PYD^ were used to assess nucleation of ASC^PYD^ filament formation by AIM2^PYD^ upon removal of MBP from both proteins (Supplementary Figure S2C).

We quantified the rate of potentiation of ASC^PYD^ filament formation, which gave an apparent AIM2^PYD^/ASC^PYD^ affinity of ~0.68 μM (Supplementary Figure S2C). Therefore, the GFP tag did not interfere with the nucleating capability of AIM2^PYD^, suggesting that ASC^PYD^ filament could also be assembled by symmetry similar to that of the GFP-AIM2^PYD^. The apparent slightly weaker interaction between untagged AIM2^PYD^ and ASC^PYD^ may be due to instability of untagged AIM2^PYD^. This tolerance of symmetry and packing variability in both AIM2^PYD^ and ASC^PYD^ is notable and may contribute to the versatility of the DD superfamily in mediating oligomerization.

To determine whether this concept of variability tolerance is more general, we produced mCherry-tagged NLRP6^PYD^ and confirmed its ability to nucleate ASC^PYD^ filaments (Supplementary Figure S2D). The N-terminal mCherry tag is of similar size as the GFP-tag, and did not disrupt the interaction between NLRP6^PYD^ and ASC^PYD^. The measured apparent affinity for mCherry-NLRP6^PYD^ to promote ASC^PYD^ filament formation is ~0.09 μM (Supplementary Figure S2D).

### Structural features of the AIM2^PYD^ filament

**Figure 3:**
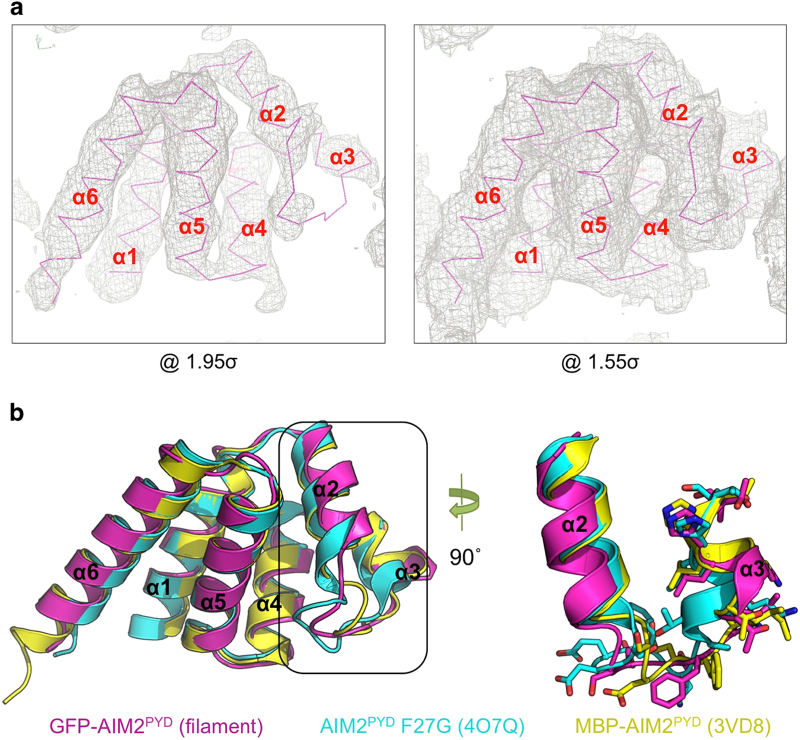
Conformational changes between AIM2^PYD^ structures in isolation and in the filament. (**a**) Cryo-EM maps at two contour levels showing that, despite the lower density in the α2-α3 region, the trajectory of the loop is defined. (**b**) Comparison of AIM2^PYD^ structure in the filament (magenta) with that in isolation (yellow) and with the F27G mutant (cyan).

We used the structure refinement program Phenix and electron scattering factors to assess the agreement between the atomic model of the filament and the cryo-EM density. The cropped cryo-EM density corresponding to 15 AIM2^PYD^ subunits that has been adjusted in real space was placed into a crystallographic unit cell and back transformed to obtain the structure factors. Upon rigid body refinement at 5.0 Å resolution with fixed B-factors for each subunit, the *R* and *R*_free_ factors were 0.43 and 0.47, respectively. Almost all the main chain density was observed, with part of the α2-α3 loop visible at a lower contour level (Figure 3a). Compared with the crystal structure of the monomeric MBP-fused wild-type AIM2^PYD^, the filament conformation of AIM2^PYD^ shows main differences at this region (Figure 3b), suggesting that AIM2^PYD^ possesses intrinsic flexibility to allow for subtle conformational changes during oligomerization. The F27G mutant of AIM2^PYD^ also exhibits differences with the wild-type AIM2^PYD^ at this region (Figure 3b).

**Figure 4:**
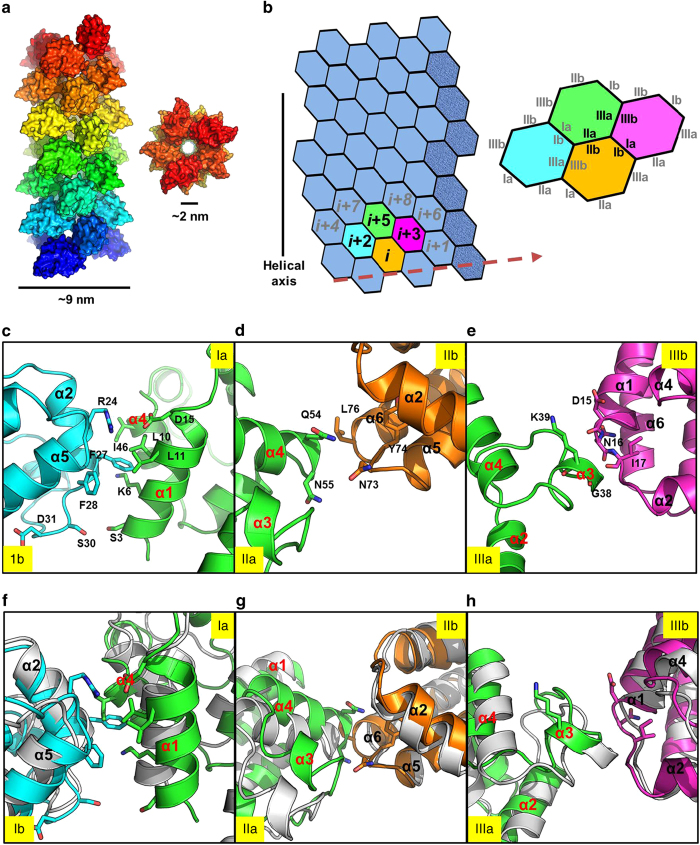
Detailed interactions in the AIM2^PYD^ filament structure. (**a**) A surface representation of the AIM2^PYD^ filament with subunits individually colored by chain in a rainbow gradient. (**b**) A schematic diagram of the helical assembly of the AIM2^PYD^ filament. The red dotted arrow indicates the direction of the helical spiral as defined by a rise of 6.0 Å and a right-handed rotation of 138.9° per subunit. It should be noted that adjacent subunits i and i+1 do not directly contact. The right panel shows locations of the three types of asymmetric interactions. (**c**–**e**) Detailed interactions in the type I (**c**), type II (**d**) and type III (**e**) interfaces. Subunit colors match those in the schematic (**b**). (**f–** **h**) AIM2^PYD^ subunits were aligned onto the ASC^PYD^ filament, and the thus generated AIM2/AIM2 interactions are shown in the same color schemes as in **c**–**e**. Gray ribbons show the corresponding AIM2/AIM2 interactions in the GFP-AIM2^PYD^ filament structure.

The AIM2^PYD^ filament core has an outer diameter of ~9 nm and an inner hole with a diameter of ~2 nm (Figure 4a), similar to dimensions of the ASC^PYD^ filament [17]. The filament has an apparent bumpy surface, in contrast to the smoother surface of the ASC^PYD^ filament due to the longer C-terminal helix in AIM2^PYD^. While the GFP-AIM2^PYD^ filament has the symmetry of a right-handed 1-start helix with 138.9° rotation and 6.0 Å axial rise per subunit, the sequential intrastrand subunits do not make direct molecular contact (Figure 4b). If we sequentially label the AIM2^PYD^ subunits according to the helical operator, the three types of interactions that dictate helical assembly in the AIM2^PYD^ filament will be between the first (i) and the fourth (i+3) subunits for the type I contacts, between the first (i) and the sixth (i+5) subunits for the type II contacts, and between the first (i) and the third (i+2) for the type III contacts (Figure 4b). Orientation and position of the three types of interactions are conserved between the AIM2^PYD^ and ASC^PYD^ structures (Figures 4c and h). Similar to ASC^PYD^, the type I interaction is the most extensive of the three types. Notably, type I interfaces in AIM2^PYD^ are mainly hydrophobic in nature, while that in ASC^PYD^ is formed by salt bridges between positively and negatively charged residues.

In the AIM2^PYD^ filament structure, the type I interaction is formed by Ser3, Lys6, Leu10, Leu11, Asp15 and Ile46 of the α1/α4 helixes of one subunit and Arg24, Phe27, Phe28 and Asp31 of the α2-α3 region of an adjacent subunit (Figure 4c). Gln54 and Asn55 at the end of the α4 helix interact with Asn73, Tyr74 and Leu76 in between α5-α6 of an adjacent molecule to form the type II contacts (Figure 4d). Type III interactions are mediated by Gly38 and Lys39 of α3 and the nearby Asp15, Asn16 and Ile17 residues of another subunit (Figure 4e). To determine whether there are significant changes in these interfacial contacts if the AIM2^PYD^ filament assumes the ASC^PYD^ filament symmetry, we superimposed the AIM2^PYD^ subunit structure onto the respective type I, II and III interaction pairs in the ASC^PYD^ filament (Figures 4f and g). This exercise shows that the orientation and position of the three types of interactions are highly similar with either the observed GFP-AIM2^PYD^ filament symmetry or the ASC^PYD^ filament symmetry (Figures 4f and g). There are minimal adjustments of the interfacial contacts, further supporting the helical net analysis (Figure 2b). Like the ASC^PYD^ filament, the type I interaction in the AIM2^PYD^ filament is the most extensive of the three types of interactions. However, the type I interface in the AIM2^PYD^ filament is mainly hydrophobic in nature, while that in the ASC^PYD^ filament is formed by salt bridges between positively and negatively charged residues.

### AIM2^HIN^ forms a filamentous complex with dsDNA

Previous studies have shown that full-length IFI16, an ALR, cooperatively assembles filaments with dsDNAs [26]. IFI16 has an N-terminal PYD followed by two HIN domains. Cooperative binding and filament formation require the PYD, while the individual HIN domains or the two HIN domains together did not show significant dsDNA binding at the close to physiological salt concentration used in the assays [26], consistent with the observed salt-dependence of IFI16 HIN in interaction with dsDNAs [13]. We asked whether AIM2 also forms filaments with dsDNAs. It has been shown previously that AIM2 activation in cells is dependent on transfected dsDNA length [10, 13]. Under one assay condition, in human peripheral blood mononuclear cells (PBMCs), an 80-bp dsDNA, in comparison with dsDNA of 50-bp or less, was a better inducer of cell death [13]. Under another condition, in mouse bone marrow-derived macrophages (BMDMs), a 44-bp dsDNA could kill mouse BMDMs when transfected at high concentrations (≥20 µg) and even longer dsDNA, 100–500 bp in lengths, induced progressively more killing when transfected [10]. In contrast, shorter dsDNA did not efficiently induce cell death [10].

**Figure 5:**
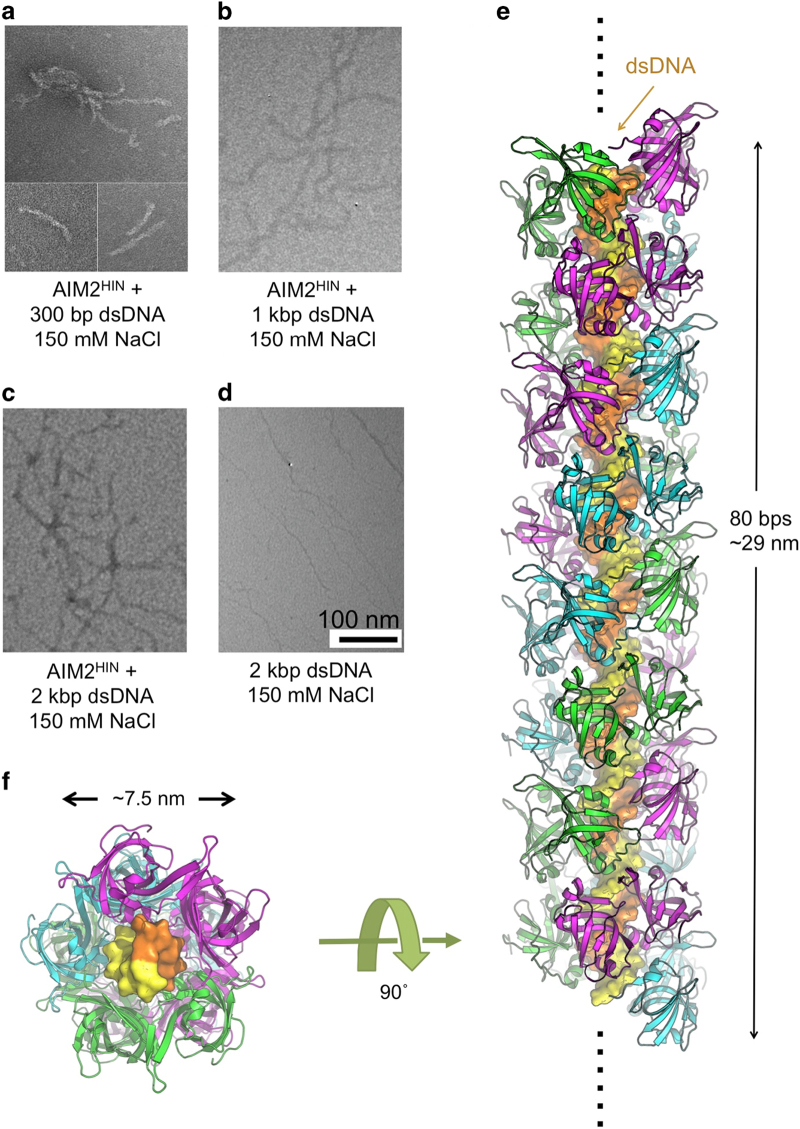
AIM2^HIN^ forms filaments with dsDNA. (**a**–**c**) EM images of negatively stained AIM2^HIN^ (0.05 mg ml^−1^) mixed with 300 bp dsDNA (**a**), 1 kbp dsDNA (**b**) and 2 kbp dsDNA (**c**). The molar ratios of AIM2^HIN^ to dsDNA are 60:1, 200:1 and 400:1, respectively, for 300 bp, 1 kbp and 2 kbp dsDNAs. (**d**) An EM image of negatively stained 2 kbp dsDNA. (**e**, **f**) A model of the AIM2^HIN^/ dsDNA filament, shown along (**e**) and down (**f**) the helical axis. The dsDNA is illustrated as a surface diagram with the two helical strands colored in orange and yellow, respectively. AIM2^HIN^ domains are colored in green, cyan and magenta.

As a 44-bp or 80-bp dsDNA is too short to be visualized reliably using negative staining electron microscopy, we used polymerase chain reaction to generate longer dsDNA fragments. We first tried AIM2^HIN^, which was expressed as a His-Sumo-fusion with the tag cleaved off during purification. In contrast to IFI16, we found that AIM2^HIN^ is able to form filaments with 300 bp, 1 kbp and 2 kbp gel-purified dsDNA fragments under close to physiological pH and salt concentrations (Figures 5a and c). The diameter of the AIM2^HIN^/dsDNA filaments is measured to be close to but <10 nm. The dsDNA alone control showed much thinner threads (Figure 5d), confirming that the filaments in the presence of AIM2^HIN^ are the AIM2^HIN^/dsDNA complexes. With increasing dsDNA lengths, the AIM2^HIN^/dsDNA filaments become curvier and more convoluted. The calculated linear lengths for 300 bp, 1 kbp and 2 kbp dsDNA fragments are 108, 360 and 720 nm, respectively.

Formation of a full-length AIM2/dsDNA complex is hampered by the insolubility of full-length AIM2 when overexpressed in *E*. *coli* as a His-Sumo-tagged protein, in contrast to the soluble nature of full-length IFI16 [26]. This difference is most likely due to the PYD domain, as the PYD of IFI16 is much less aggregated than AIM2^PYD^ when expressed alone (Supplementary Figure S3). Unlike AIM2 that was shown to be auto-inhibited [13, 16], IFI16 appears to exist in an extended conformation in the absence of dsDNA stimulation [11]. Overexpression of full-length AIM2 may relieve this auto-inhibition and therefore leads to aggregation and insolubility. To overcome this difficulty, we expressed AIM2 as a fusion to the large solubility tag His-MBP to inhibit self-oligomerization. Purified monomeric His-MBP-AIM2 was then mixed with dsDNA, to which the TEV protease was added to remove the His-MBP tag. In contrast to formation of IFI16/dsDNA filaments [26], full-length AIM2 precipitated upon encountering dsDNA and TEV, suggesting large-scale aggregation upon activation. This apparent higher aggregation tendency of AIM2 than IFI16 may correlate with the need to sense small amounts of dsDNA in the cytosol.

To model the AIM2^HIN^/dsDNA filament complex, we used an existing human AIM2^HIN^/dsDNA complex crystal structure (PDB ID: 3RN5) [13]. We used 80 bps as one optimal dsDNA length for AIM2 activation, and first aligned one complex to an ideal 80-bp B-form dsDNA by superimposing the dsDNA molecules. We reasoned that a most plausible way to model a dsDNA coated with AIM2^HIN^ is by aligning the same complex to different segments in the 80 bp dsDNA. We attempted different DNA step sizes. Remarkably, a step size of every 4 bps generated an AIM2^HIN^/dsDNA filament complex in which the AIM2^HIN^ molecules are in extensive contact, but with no significant steric clash (Figure 5e). In contrast, a step of 3 or 5 bps generated AIM2^HIN^ molecules on dsDNA that are either in severe clash or with no contact, suggesting that the 4 bps step is optimal. Assuming that each HIN domain occupies 4 bps, the molar ratio of protein to DNA-binding sites would have been 0.8:1 for all the EM experiments (Figures 5a and c). In this predicted AIM2^HIN^/dsDNA filament model, each HIN molecule is in contact with six adjacent HINs. The diameter of the model with dsDNA decorated by an outer layer of AIM2^HIN^ molecules is ~7.5 nm (Figure 5f), consistent with the EM observation. The ~50-residue linker between the PYD and HIN domains may allow the PYDs to swing around the dsDNA core to form a short helical assembly of AIM2^PYD^ molecules for interacting with and nucleating ASC^PYD^ filaments.

## Discussion

AIM2 is a critically important cytoplasmic dsDNA sensor that activates caspase-1 to provide host defense against pathogens. Here we present the cryo-EM structure of the PYD domain of AIM2 in its activated, filamentous form. The cryo-EM images and the low-resolution power spectrum are both dominated by the GFP tag, which is poorly ordered. Despite this difficulty, we were able to obtain a map of the central AIM2^PYD^ filament at a resolution that is clearly interpretable.

We and others have previously identified numerous AIM2^PYD^ mutants that are defective in their abilities to self-aggregate and/or to nucleate ASC^PYD^ filaments [16, 17, 19] (Supplementary Figure S4A). Strikingly, when these mutation sites are mapped onto the AIM2^PYD^ structure, almost all mutations are exactly at the observed interfacial contacts in the AIM2^PYD^ filament structure (Supplementary Figure S4B). The type I interface AIM2^PYD^ mutants F27G, F27L, L10A/L11A and D31K did not self-aggregate [19]. F27G, L10A/L11A and R24E mutations abolished the ability of AIM2^PYD^ to nucleate ASC^PYD^ filaments [17]. The type II mutant, Y74R, lost the ability to nucleate ASC^PYD^ [17]. Located at the edge of both type Ia and type IIIb interfaces, D15R was also completely defective. Type III mutants, G38E and K39E, appeared to have partially lost their ability to interact with ASC^PYD^. The quadruple mutant D19A/E20A/E21A/D23A near the type Ib interface also showed compromised ability to interact with ASC^PYD^ [16]. These existing mutational data confirmed the validity of the observed interactions in the AIM2^PYD^ filament. Human AIM2^PYD^ and IFI16^PYD^ share 31% sequence identity. Sequence alignment of IFI16^PYD^ to AIM2^PYD^ shows that the interfacial residues important for AIM2^PYD^ filament formation and function are mostly conserved in IFI16^PYD^ (Supplementary Figure S4C), suggesting that IFI16^PYD^ uses a similar assembly mechanism for initiating inflammasome formation and signaling.

**Figure 6:**
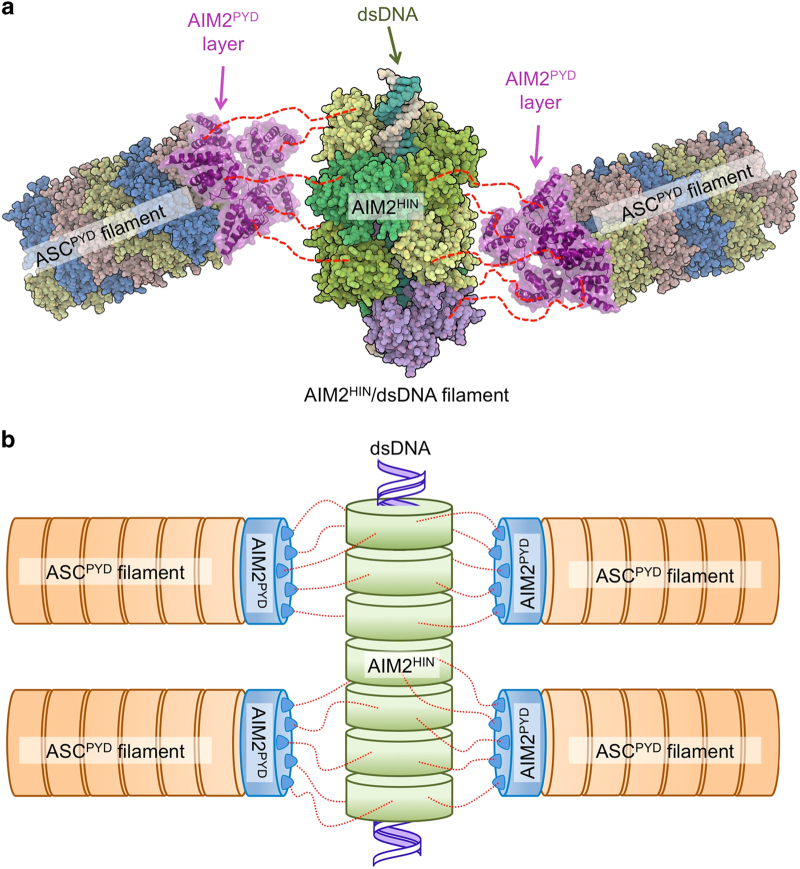
A conceptual model of ASC^PYD^ nucleation by the AIM2/dsDNA complex, shown as an atomic model (**a**) and a schematic (**b**).

Our structure and the EM analysis on AIM2^HIN^/dsDNA interaction provide a conceptual model in dsDNA-induced AIM2 activation (Figures 6a and b). First, cytoplasmic dsDNA of sufficient length from invading pathogens will recruit AIM2 through HIN/dsDNA interactions. Wrapping of the HIN domain around the dsDNA not only overcomes AIM2 auto-inhibition, but also brings the attached AIM2^PYD^ into proximity to form helical assemblies. There is a fairly long, ~50-residue linker between the PYD and the HIN, which can be as long as ~19 nm when completely stretched. As the AIM2^HIN^/dsDNA filament is ~7.5 nm in diameter, it takes ~12 nm to reach from one side of the filament to the opposing side, shorter than what the linker could theoretically reach. The AIM2^PYD^ filaments in turn act as the platforms to nucleate ASC^PYD^ filaments, resulting in inflammasome assembly and caspase-1 activation. How exactly AIM2^PYD^ assembles on the side of dsDNA still remains to be illustrated. The linker between HIN and PYD, albeit long, may impose a spatial restrain on the length of the PYD helical assembly, and promote formation of short local AIM2^PYD^ filaments. Despite the likelihood of being short, the helical nature of AIM2^PYD^ on the side of dsDNA is supported by previous mutagenesis data and cellular activation assays, which showed the importance of the interfacial residues in the AIM2^PYD^ helical assembly for activation of the AIM2 inflammasome [1, 17, 19].

The significantly less-dense packing in the GFP-AIM2^PYD^ filament relative to the ASC^PYD^ filament suggests plasticity in PYD filament assembly. Although the GFP fused to AIM2^PYD^ via a mere two-residue linker most likely affected the symmetry of the filament, the much longer PYD-HIN linker would allow AIM2^PYD^ to assume its natural helical symmetry without being perturbed by the attached HIN domain. The HIN domain is of similar size to GFP. One possibility is that AIM2^PYD^ does have a natural symmetry identical to ASC^PYD^. Another possibility is that AIM2^PYD^ forms filaments using a symmetry somewhat different from ASC^PYD^. Conversely, ASC^PYD^ may be able to assume different symmetries depending on which binding partner it interacts with. It is worth noting that ALRs and most members of the large NLR family have a PYD, which use the single adaptor ASC for inflammasome formation. The minimal changes in subunit interactions in an altered symmetry should allow successful PYD/PYD interaction despite the symmetry difference. Therefore, the observed structural plasticity must be important for the functional requirement of ASC in interacting with multiple PYDs in inflammasome formation. In addition, the AIM2^PYD^ filament structure reported here represents the first case in which adaptability has been observed for the entire superfamily of DD-containing proteins.

Many other filamentous systems have also been known to possess variability. For example, tubulin can be polymerized *in vitro* to form microtubules with 9 to 15 or more protofilaments, and transitions between these can be seen in the same microtubule [27]. F-actin possesses considerably variable and randomized twist but a relatively constant rise per subunit [28]. The DD fold superfamily represents the most abundant domain in innate immune signaling pathways. These domains are versatile modules for both homotypic and heterotypic molecular interactions that afford the scaffolds for their respective signaling complexes. Given the conserved mode of interactions in the DD fold superfamily, this observed plasticity in PYD assembly may contribute to the adaptability of the DD fold in general, and likely explain the omnipresence of these domains in innate immune pathways.

## Materials and Methods

### Construct and filament sample preparation

To generate a His-GFP-tagged AIM2^PYD^, the cDNA of monomeric GFP (M1-T230, A206K) was cloned into the pET28a backbone using the *Nde*I and *Bam*HI sites. AIM2^PYD^ (M1-P100) was inserted in frame with the *GFP* gene using *Bam*HI and *Not*I sites, which introduced a two-residue linker by the *Bam*HI site. The construct was expressed in *E. coli* BL21(DE3) cells by overnight induction using IPTG at 18˚C. The cells were collected and lysed in lysis buffer containing 20 mM HEPES pH 8.0, 200 mM NaCl, 5 mM imidazole, 5 mM β-mercaptoethanol (β-ME) and 10% glycerol. Following sonication, the cell debris was centrifuged at 30 000 *g* for 30 min. The supernatant after centrifugation was incubated with the Ni-NTA affinity resin for 1 h, and washed in lysis buffer containing 20 mM imidazole. His-GFP-AIM2^PYD^ filaments were eluted using lysis buffer with 300 mM imidazole. The eluate was injected onto a Superdex 200 10/300 GL column pre-equilibrated with gel filtration buffer containing 20 mM HEPES pH 8.0, 150 mM NaCl and 2 mM dithiothreitol. The filaments eluted in the void, with peak at ~8 ml. The sample was frozen in small aliquots for subsequent analysis.

### Cryo-EM imaging, indexing and reconstruction

The sample (3 μl) was applied to lacey carbon grids that were plasma cleaned (Gatan Solarus) and vitrified in a Vitrobot Mark IV (FEI, Hillsboro, OR, USA). Grids were imaged in a Titan Krios at 300 keV, and recorded with a Falcon II direct electron detector at 1.15 Å per pixel (px). A total of 207 images (each 4 k ×4 k) were selected that were free from drift or astigmatism. The program CTFFIND3 [29] was used for determining the contrast transfer function and the defocus range used was from 0.6 to 5.0 μm. The SPIDER software package [30] was used for most subsequent steps. The contrast transfer function was corrected by multiplying each image with the theoretical contrast transfer function, both reversing phases where they need to be reversed and improving the signal-to-noise ratio. The program e2helixboxer within EMAN2 [31] was used for boxing long filaments from the micrographs. Overlapping boxes, 384 px long with a 10 px shift between adjacent boxes (97% overlap) were extracted from these long filaments, yielding 54 973 segments.

Starting with an averaged power spectrum, it was apparent that many different helical symmetries were possible [22]. The correct helical symmetry was determined by trial-and-error, searching for a solution using the iterative helical real space reconstruction method [23] that yielded recognizable secondary structure [24]. The symmetry search was complicated by the fact that out-of-plane tilt cannot be ignored [24], and each symmetry that was tried needed to allow for such out-of-plane tilt. The symmetry that was found, a rise of 6.0 Å and a rotation of 138.9° per subunit, generated a reconstruction where the PYD was seen to be largely α-helical, and was thus taken to be correct.

### AIM2 model fitting and refinement

The EM density was first transformed to CCP4 format [32]. A small region corresponding to the density of a single AIM2^PYD^ molecule was cutoff with a mask, and wild-type AIM2^PYD^ structure (3VDB) was aligned to the density. Helices were moved slightly by fitting individually in real space and regularized in Coot to generate an AIM2^PYD^ structure in the filament conformation [25]. The modified AIM2^PYD^ structure was fitted individually into EM density in real space to generate an initial AIM2^PYD^ filament structure with 15 molecules. The density corresponding to the 15-molecule filament was cut using a mask generated from the model and put into an arbitrary unit cell in the P1 space group. Structure factors were calculated using electron scattering factors from the resulted map, and the figure of merit and the sigma were added to each reflection uniformly. Structure refinement was carried with PHENIX.refine [33], using rigid body positional refinement and a single B-factor for each subunit. The refinement led to an *R* of 0.43, *R*_free_ of 0.47, root mean square deviation in bond lengths of 0.016 Å and root mean square deviation in bond angles of 0.48°.

### Fluorescence polarization assay

Detailed protocol could be found in our previous study [17]. In short, monomeric MPB-tagged ASC^PYD^ (M1-Q105) containing an engineered Cys at the C-terminus (S106C) was expressed in *E*. *coli* BL21(DE3) cells and purified by Ni-NTA affinity pulldown and SEC. The monomers were labeled with Maleimide-Alexa Fluor 568 (Invitrogen, Grand Island, NY, USA) by recommended protocol from the manufacturer. Excess dyes were removed by gel filtration after overnight incubation. Fluorescence polarization measurements were taken with the SpectraMax M5e (Molecular Devices, Sunnyvale, CA, USA) plate reader. For the mCherry-NLRP6^PYD^/ASC^PYD^ experiment, same procedure was used except Maleimide-Alexa Fluor 488 was used to label MBP-ASC^PYD^.

### Negative-stain EM analysis of AIM2^HIN^/dsDNA filaments

AIM2^HIN^ (residues 138–343) was cloned into pSMT3 vector using the *Bam*HI and *Sal*I cloning sites and expressed as a Sumo-fusion protein with an additional N-terminal 6× His tag. The recombinant protein was expressed in *E*. *coli* BL21(DE3) cells by growing the culture at 37 °C to OD_600_ of 0.8 and inducing with 0.5 mM IPTG overnight at 16 °C. The *E*. *coli* cells were harvested and lysed by sonication in lysis buffer containing 25 mM Tris-HCl pH 8.0, 1.0 M NaCl, 5 mM imidazole, 5 mM β-ME and 5% glycerol. The cell lysate was centrifuged at 40 000 *g* for 40 min, and the supernatant was incubated with pre-equilibrated Ni-NTA resin. After being washed with 20 column-volume lysis buffer containing 25 mM imidazole, the protein was eluted in buffer A containing 25 mM Tris-HCl pH 8.0, 300 mM NaCl, 300 mM imidazole, 5 mM β-ME and 5% glycerol. To cleave the Sumo-tag, the recombinant protein was incubated with 1/1 000 (w/w) Ulp1 protease at 4 °C overnight. The cleaved protein was then loaded onto a Heparin SP column and eluted with a gradient of 0.3–1.5 M NaCl in buffer A. The peak corresponding to AIM2^HIN^ was further purified using Superdex 200 (10/30) SEC in 25 mM Tris-HCl pH 8.0, 150 mM NaCl and 2 mM DTT. Fractions containing AIM2^HIN^ were collected, concentrated and flash frozen in liquid nitrogen for future use. To form AIM2^HIN^/dsDNA filaments, AIM2^HIN^ was diluted in SEC buffer to a final concentration of 0.05 mg ml^−1^, and incubated with 300 bp, 1 kbp and 2 kbp dsDNA at a molar ratio of 1/60, 1/200 and 1/400 of the protein, respectively.

Full-length AIM2 was cloned into pDB.His.MBP vector using the *Nde*I and *Xho*I cloning sites and expressed as a His-MBP-fusion protein in *E*. *coli* BL21(DE3) cells under the same condition as that used for AIM2^HIN^. The protein was purified with Ni-NTA resin followed by Heparin SP and Superdex 200 (10/30) columns without removing the MBP-tag. The purified MBP-AIM2 protein was diluted to 0.05 mg ml^−1^ and mixed with dsDNA of different lengths at indicated concentrations, and TEV protease was added to 1/10 (w/w) ratio with the protein to cleave the MBP-tag, allowing for AIM2/dsDNA filament formation.

### Modeling the AIM2^HIN^/dsDNA filament

An 80-bp ideal B-form DNA (DNA80) is generated in WinCoot [25]. Superimposing the AIM2^HIN^/dsDNA structure (PDB ID: 3RN5) on DNA80 generated the first HIN domain on DNA80. Sliding the first HIN domain along DNA80 every four base pairs (i → i+4) results in HIN domains closely packed on DNA80 without significant clashes between adjacent molecules.

## References

[bib1] Lamkanfi M , Dixit VM . Mechanisms and functions of inflammasomes. Cell 2014; 157: 1013–1022. 24855941 10.1016/j.cell.2014.04.007

[bib2] Lu A , Wu H . Structural mechanisms of inflammasome assembly. FEBS J 2014; 282: 435–444. 25354325 10.1111/febs.13133 PMC6400279

[bib3] Hu Z , Yan C , Liu P , et al. Crystal structure of NLRC4 reveals its autoinhibition mechanism. Science 2013; 341: 172–175. 23765277 10.1126/science.1236381

[bib4] Ting JP , Lovering RC , Alnemri ES , et al. The NLR gene family: a standard nomenclature. Immunity 2008; 28: 285–287. 18341998 10.1016/j.immuni.2008.02.005 PMC2630772

[bib5] Kerur N , Veettil MV , Sharma-Walia N , et al. IFI16 acts as a nuclear pathogen sensor to induce the inflammasome in response to Kaposi Sarcoma-associated herpesvirus infection. Cell Host Microb 2011; 9: 363–375. 10.1016/j.chom.2011.04.008 PMC3113467 21575908

[bib6] Rathinam VA , Jiang Z , Waggoner SN , et al. The AIM2 inflammasome is essential for host defense against cytosolic bacteria and DNA viruses. Nat Immunol 2010; 11: 395–402. 20351692 10.1038/ni.1864 PMC2887480

[bib7] Hornung V , Ablasser A , Charrel-Dennis M , et al. AIM2 recognizes cytosolic dsDNA and forms a caspase-1-activating inflammasome with ASC. Nature 2009; 458: 514–518. 19158675 10.1038/nature07725 PMC2726264

[bib8] Fernandes-Alnemri T , Yu JW , Datta P , Wu J , Alnemri ES . AIM2 activates the inflammasome and cell death in response to cytoplasmic DNA. Nature 2009; 458: 509–513. 19158676 10.1038/nature07710 PMC2862225

[bib9] Burckstummer T , Baumann C , Bluml S , et al. An orthogonal proteomic-genomic screen identifies AIM2 as a cytoplasmic DNA sensor for the inflammasome. Nat Immunol 2009; 10: 266–272. 19158679 10.1038/ni.1702

[bib10] Roberts TL , Idris A , Dunn JA , et al. HIN-200 proteins regulate caspase activation in response to foreign cytoplasmic DNA. Science 2009; 323: 1057–1060. 19131592 10.1126/science.1169841

[bib11] Liao JC , Lam R , Brazda V , et al. Interferon-Inducible Protein 16: Insight into the Interaction with Tumor Suppressor p53. Structure 2011; 19: 418–429. 21397192 10.1016/j.str.2010.12.015 PMC3760383

[bib12] Theobald DL , Mitton-Fry RM , Wuttke DS . Nucleic acid recognition by OB-fold proteins. Annu Rev Biophys Biomol Struct 2003; 32: 115–133. 12598368 10.1146/annurev.biophys.32.110601.142506 PMC1564333

[bib13] Jin T , Perry A , Jiang J , et al. Structures of the HIN domain:DNA complexes reveal ligand binding and activation mechanisms of the AIM2 inflammasome and IFI16 receptor. Immunity 2012; 36: 561–571. 22483801 10.1016/j.immuni.2012.02.014 PMC3334467

[bib14] Ru H , Ni X , Zhao L , et al. Structural basis for termination of AIM2-mediated signaling by p202. Cell Res 2013; 23: 855–858. 23567559 10.1038/cr.2013.52 PMC3674390

[bib15] Sung MW , Watts T , Li P . Crystallographic characterization of mouse AIM2 HIN-200 domain bound to a 15 bp and an 18 bp double-stranded DNA. Acta Crystallogr Sect F Struct Biol Cryst Commun 2012; 68: 1081–1084. 10.1107/S174430911203103X PMC3433203 22949200

[bib16] Jin T , Perry A , Smith P , Jiang J , Xiao TS . Structure of the absent in melanoma 2 (AIM2) pyrin domain provides insights into the mechanisms of AIM2 autoinhibition and inflammasome assembly. J Biol Chem 2013; 288: 13225–13235. 23530044 10.1074/jbc.M113.468033 PMC3650362

[bib17] Lu A , Magupalli VG , Ruan J , et al. Unified polymerization mechanism for the assembly of ASC-dependent inflammasomes. Cell 2014; 156: 1193–1206. 24630722 10.1016/j.cell.2014.02.008 PMC4000066

[bib18] Yin Q , Sester DP , Tian Y , et al. Molecular mechanism for p202-mediated specific inhibition of AIM2 inflammasome activation. Cell Rep 2013; 4: 327–339. 23850291 10.1016/j.celrep.2013.06.024 PMC3760141

[bib19] Lu A , Kabaleeswaran V , Fu T , Magupalli VG , Wu H . Crystal structure of the F27G AIM2 PYD mutant and similarities of its self-association to DED/DED interactions. J Mol Biol 2014; 426: 1420–1427. 24406744 10.1016/j.jmb.2013.12.029 PMC3951648

[bib20] Cai X , Chen J , Xu H , et al. Prion-like polymerization underlies signal transduction in antiviral immune defense and inflammasome activation. Cell 2014; 156: 1207–1222. 24630723 10.1016/j.cell.2014.01.063 PMC4034535

[bib21] Ferrao R , Wu H . Helical assembly in the death domain (DD) superfamily. Curr Opin Struct Biol 2012; 22: 241–247. 22429337 10.1016/j.sbi.2012.02.006 PMC3320699

[bib22] Egelman EH . Reconstruction of helical filaments and tubes. Methods Enzymol 2010; 482: 167–183. 20888961 10.1016/S0076-6879(10)82006-3 PMC3245864

[bib23] Egelman EH . A robust algorithm for the reconstruction of helical filaments using single-particle methods. Ultramicroscopy 2000; 85: 225–234. 11125866 10.1016/s0304-3991(00)00062-0

[bib24] Egelman EH . Ambiguities in helical reconstruction. eLife 2014; 3: 1–9. 10.7554/eLife.04969 PMC4371874 25486515

[bib25] Emsley P , Cowtan K . Coot: model-building tools for molecular graphics. Acta Crystallogr D Biol Crystallogr 2004; 60: 2126–2132. 15572765 10.1107/S0907444904019158

[bib26] Morrone SR , Wang T , Constantoulakis LM , Hooy RM , Delannoy MJ , Sohn J . Cooperative assembly of IFI16 filaments on dsDNA provides insights into host defense strategy. Proc Natl Acad Sci USA 2014; 111: E62–E71. 24367117 10.1073/pnas.1313577111 PMC3890864

[bib27] Chretien D , Metoz F , Verde F , Karsenti E , Wade RH . Lattice defects in microtubules: protofilament numbers vary within individual microtubules. J Cell Biol 1992; 117: 1031–1040. 1577866 10.1083/jcb.117.5.1031 PMC2289483

[bib28] Egelman EH , Francis N , DeRosier DJ . F-actin is a helix with a random variable twist. Nature 1982; 298: 131–135. 7201078 10.1038/298131a0

[bib29] Mindell JA , Grigorieff N . Accurate determination of local defocus and specimen tilt in electron microscopy. J Struct Biol 2003; 142: 334–347. 12781660 10.1016/s1047-8477(03)00069-8

[bib30] Frank J , Radermacher M , Penczek P , et al. SPIDER and WEB: processing and visualization of images in 3D electron microscopy and related fields. J Struct Biol 1996; 116: 190–199. 8742743 10.1006/jsbi.1996.0030

[bib31] Tang G , Peng L , Baldwin PR , et al. EMAN2: an extensible image processing suite for electron microscopy. J Struct Biol 2007; 157: 38–46. 16859925 10.1016/j.jsb.2006.05.009

[bib32] Collaborative Computational Project N. The CCP4 Suite: Programs for Protein Crystallography. Acta Cryst 1994; D50: 760–763. 10.1107/S0907444994003112 15299374

[bib33] Adams PD , Afonine PV , Bunkoczi G , et al. PHENIX: a comprehensive Python-based system for macromolecular structure solution. Acta Crystallogr D Biol Crystallogr 2010; 66: 213–221. 20124702 10.1107/S0907444909052925 PMC2815670

