# Supplementary material for: Plasticity in PYD assembly revealed by cryo-EM structure of the PYD filament of AIM2
Source: Cell Discov. 2015 Jun 23;1:15013–. doi: 10.1038/celldisc.2015.13 (PMC4646227; doi:10.1038/celldisc.2015.13)
Supplement: Supplementary Information [file celldisc201513-s1.doc]

**Supplementary Information**


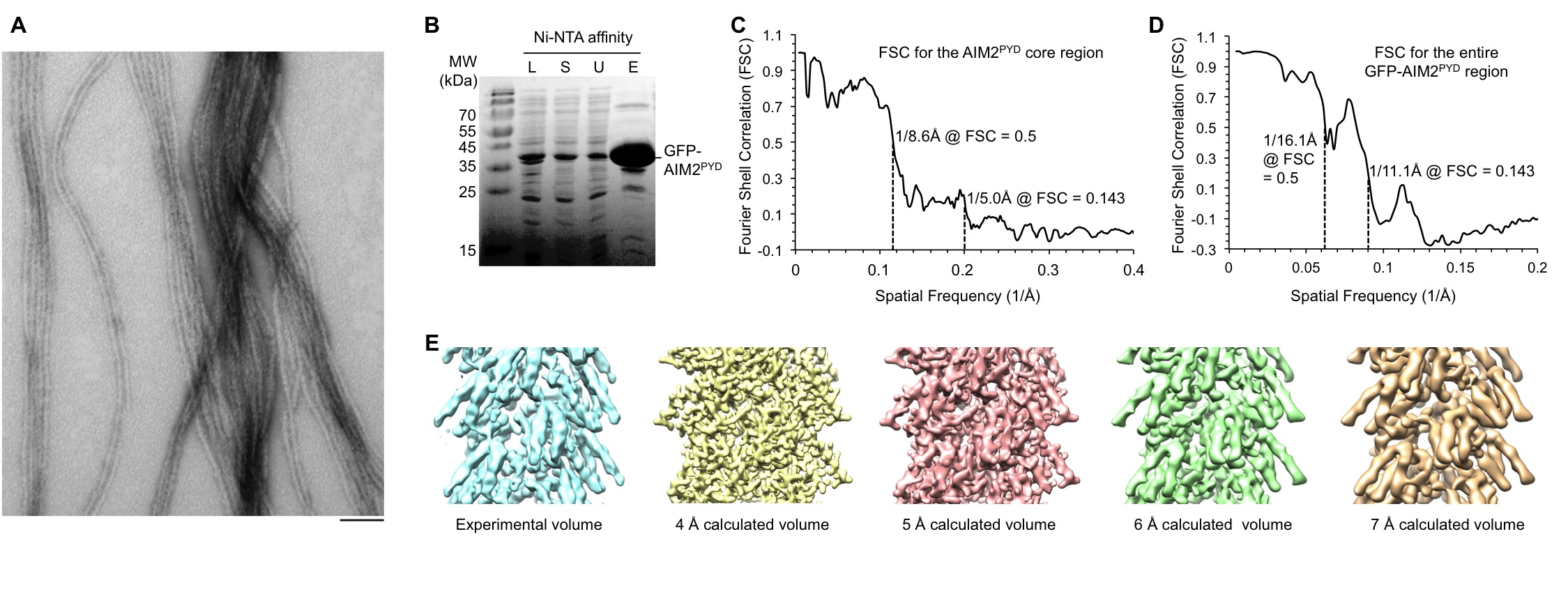


**Figure S1**. Cryo-EM structure determination of the GFP-AIM2^PYD^ filament. (**A**) Negative-stain electron micrograph of the bundled AIM2^PYD^ filaments after MBP tag removal. Scale bar at 100nm. (**B**) Ni-NTA affinity purification of the GFP-tagged AIM2­^PYD^ filament. L: lysate; S: supernatant; U: unbound fraction; E: elution. (**C**) Fourier shell correlation (FSC) plot of the AIM2^PYD^ filament core of the reconstruction. (**D**) Fourier shell correlation (FSC) plot of the entire GFP-AIM2­^PYD^ filament reconstruction. (**E**) Comparison of the experimental volume to model volumes filtered to 4, 5, 6 and 7 Å resolutions.


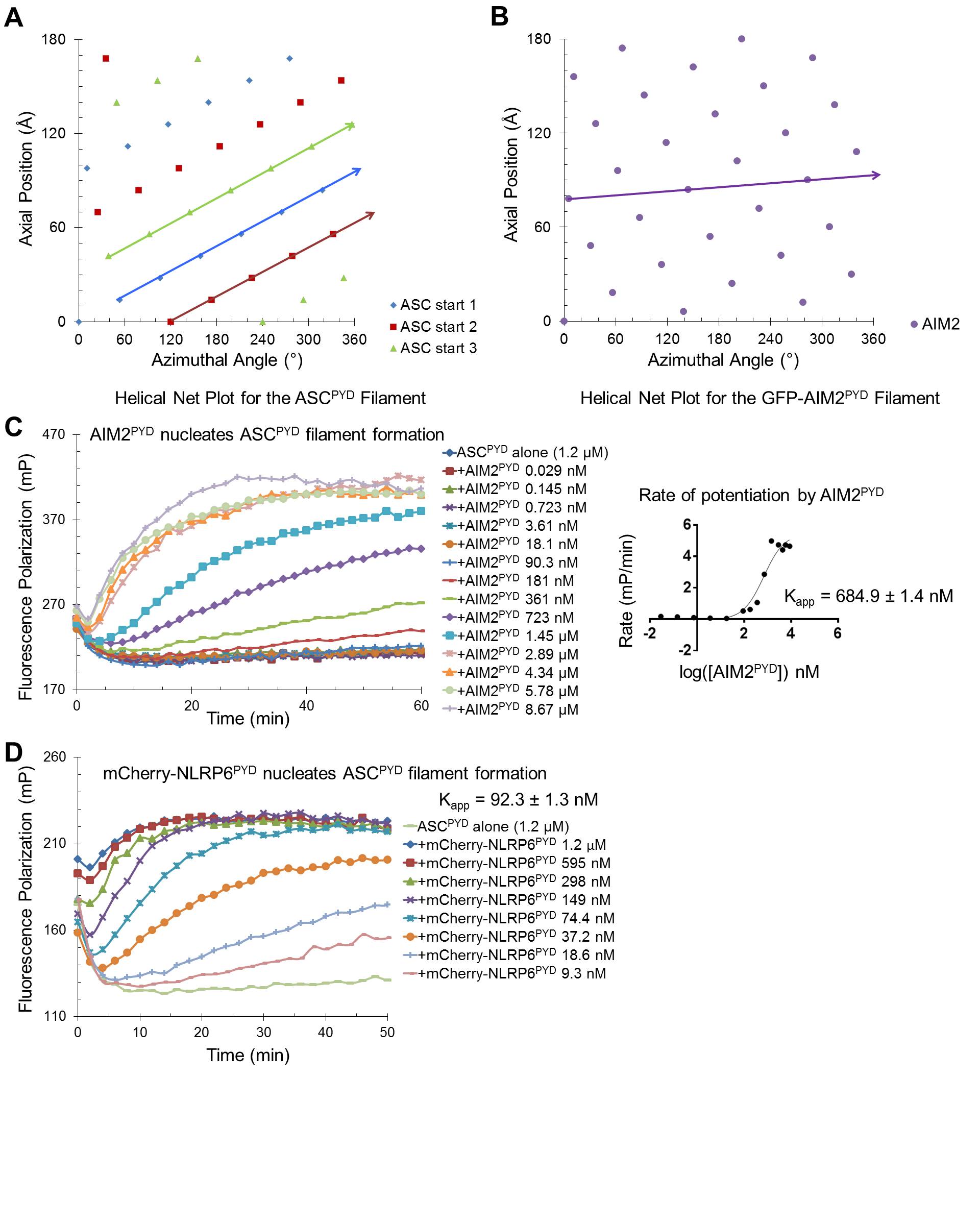


**Figure S2**. Comparison of the ASC^PYD^ filament (**A**) and the GFP-AIM2^PYD^ filament (**B**) helical net plots. Nucleation of ASC^PYD^ filament formation by AIM2^PYD^ (**C**) and mCherry-tagged NLRP6^PYD^ (**D**). The initial slopes of the nucleation experiments were extracted to estimate apparent affinity by fitting to a three-parameter agonist model.

**
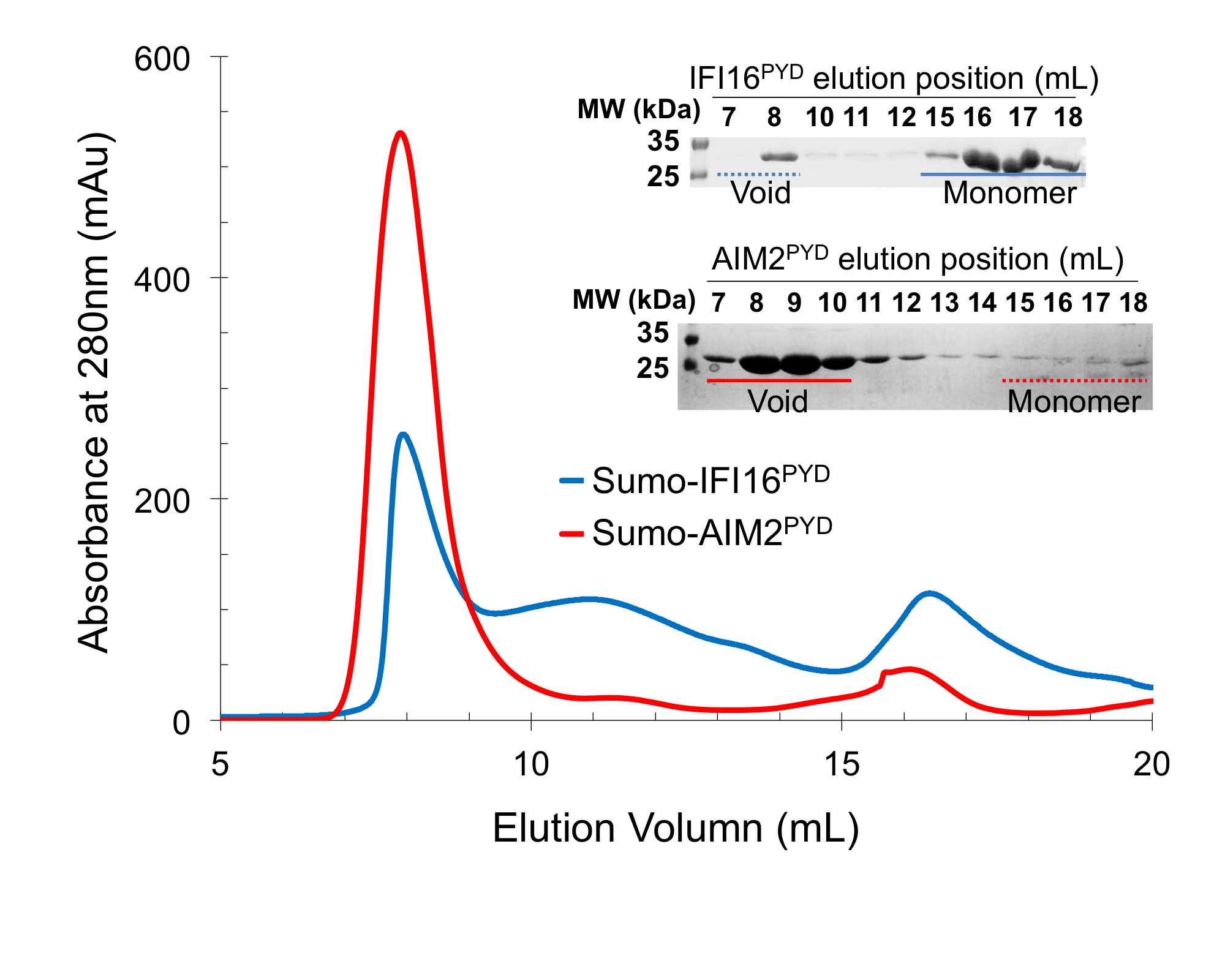
**

**Figure S3**. Comparison of the gel filtration profiles of Sumo-tagged IFI16^PYD^ and Sumo-tagged AIM2^PYD^

**
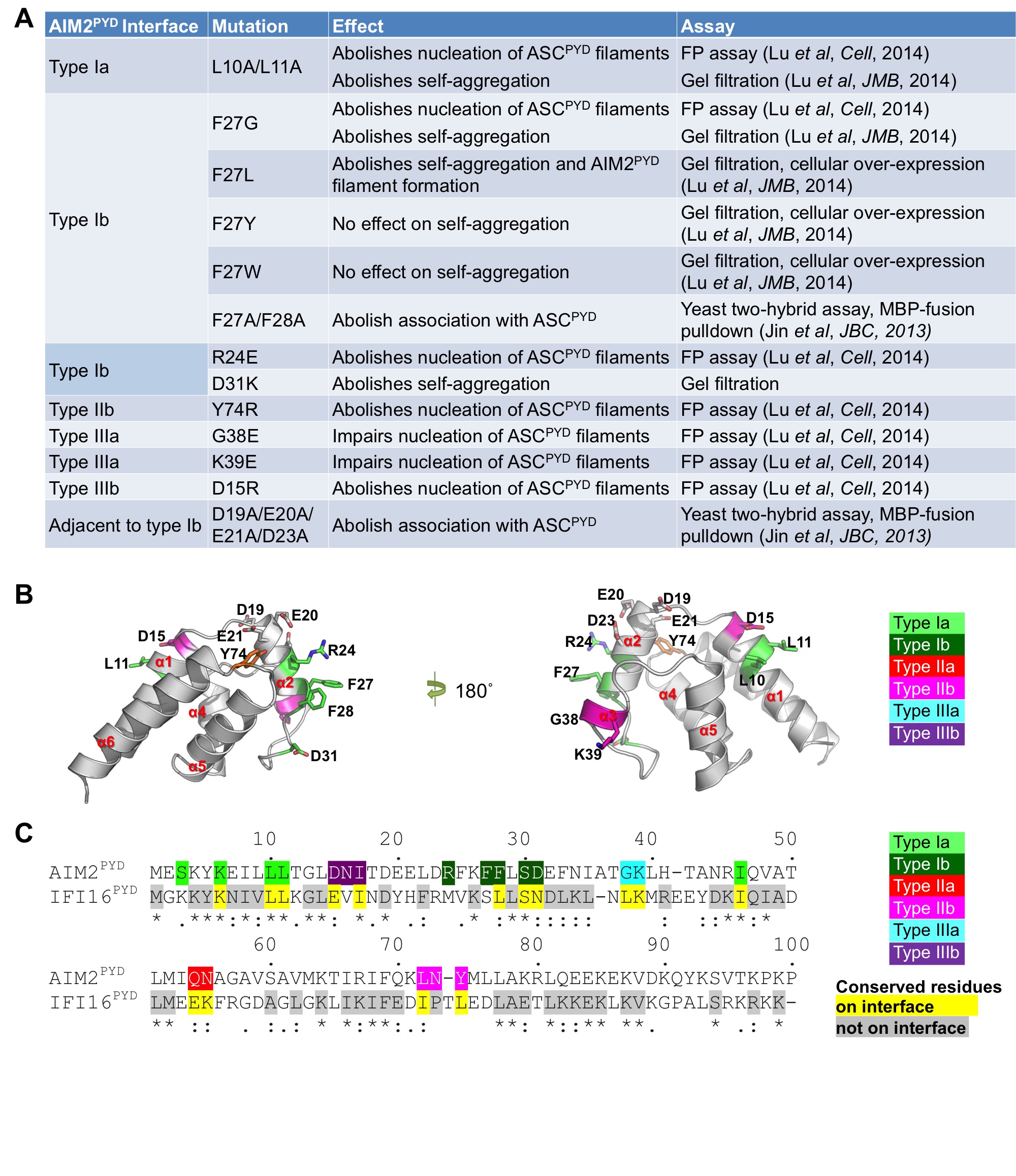
**

**Figure S4.** Mutational and sequence analysis of AIM2^PYD^. (**A**) Summary of existing mutagenesis data, with the locations of these mutated residues designated by the three types. (**B**). Mapping of the mutated residues onto the AIM2^PYD^ structure. (**C**) Sequence alignment between AIM2 and IFI16 PYDs. Interfacial residues of AIM2^PYD^ in the filament are highlighted by their interaction types. Residues of IFI16^PYD^ that are conserved with those at the AIM2^PYD^ filament interface are highlighted in yellow.

**Video 1**: A movie showing rotations along the helical axis of the experimental cryo-EM density fitted with the crystal structure of GFP and partially refined AIM2^PYD^ model.
